# Role of the NOD1/Rip2 Signaling Pathway in Macrophage Inflammatory Activation Induced by ox-LDL

**DOI:** 10.1155/crp/7601261

**Published:** 2024-11-28

**Authors:** Liang Hou, Jinli Liu, Yuhui Yuan, Yanchun Ding

**Affiliations:** ^1^Department of Cardiology, General Hospital of the Yangtze River Shipping, Wuhan, Hubei, China; ^2^Second Cardiology Department, Second Affiliated Hospital of Dalian Medical University, Dalian, China; ^3^Cancer Center, Dalian Medical University, Dalian, China

**Keywords:** atherosclerosis, macrophage polarization, NOD1, ox-LDL, Rip2

## Abstract

**Aim:** This study aimed to investigate the impact of the NOD1/Rip2 signaling pathway on macrophage inflammatory activation and polarity switching in ox-LDL-induced THP-1-derived macrophages.

**Methods:** THP-1-derived macrophages were stimulated with various concentrations (10, 25, or 50 mg/L) of ox-LDL for different durations (8, 16, or 24 h). Quantitative real-time PCR was used to measure the mRNA expression of NOD1, Rip2, IL-10, IL-12, iNOS, and Arg-1. Western blotting was used to determine the protein levels of NOD1 and Rip2. The secretion of TNF-α and MCP-1 in the cell culture supernatants was measured via ELISA. Rip2 siRNA was used to inhibit the NOD1/Rip2 signaling pathway. Oil Red O staining was employed to visualize foam cell formation. CD86, CD80, and CD163 membrane molecules were analyzed via FACS.

**Results:** After exposure to ox-LDL, the expression levels of NOD1 and Rip2 mRNAs and proteins in THP-1-derived macrophages increased in a dose- and time-dependent manner. This upregulation was accompanied by increased concentrations of TNF-α and MCP-1 in the cell culture supernatants. The effects of NOD1 and Rip2 expression upregulation were mitigated by Rip2 siRNA, as evidenced by decreased concentrations of TNF-α and MCP-1. Furthermore, ox-LDL downregulated the expression of M2 macrophage markers CD163, IL-12, and Arg-1 and upregulated the expression of M1 macrophage markers CD86, CD80, IL-10, and iNOS. The inhibition of Rip2 by siRNA reversed these effects and prevented the formation of foam cells.

**Conclusion:** Our data show that the NOD1/RIP2 signaling pathway regulates the inflammatory activation of macrophages induced by ox-LDL and controls the macrophage polarity switch.

## 1. Introduction

Coronary artery disease (CAD) is the leading cause of death worldwide. The main pathogenesis of CAD is atherosclerosis in the coronary arteries, leading to stenosis or blockage of the lumen, resulting in myocardial ischemia, hypoxia, or necrosis. Increasing evidence suggests that inflammation plays a crucial role in the formation and evolution of atherosclerotic plaques. Colchicine is an ancient anti-inflammatory drug used to treat various rheumatic conditions. Given the significant role of inflammation in the development of cardiovascular diseases, researchers have reported that the use of low-dose colchicine reduces the risk of ischemic events in patients with CAD, particularly in terms of repeated revascularizations, new myocardial infarctions, and strokes [1, 2]. Several epidemiological studies have indicated that nutraceuticals are rich in antioxidants and have anti-inflammatory, hypolipidemic, and hypoglycemic properties thought to ameliorate the progression of CAD, thus serving as potential treatments [3, 4]. Furthermore, statins, drugs widely used to treat CAD, have been demonstrated to reduce inflammatory responses and regulate the secretion of inflammatory factors [5]. These findings further confirm the potential value of exploring inflammatory mechanisms in the treatment of CAD.

Monocytes differentiate into tissue macrophages, which take up oxidized low-density lipoprotein (ox-LDL) and form foam cells. Activated macrophages can release inflammatory cytokines, chemokines, and various activators and inhibitors of proteinases, linking innate immunity to acquired immunity, regulating the accumulation of lipids, remodeling blood vessels, and stabilizing atheromas [6–9]. Macrophage inflammatory activity and polarization-switching processes involve complex signaling networks. The nucleotide-binding oligomerization domain (NOD)–like receptor, a crucial pattern recognition receptor family member, regulates the innate immune response. NOD1 and NOD2 are prominent members of the NOD family. After NOD1 or NOD2 binds to their ligands, they can recruit and activate Rip2, mediating NF-*κ*B and MAPK signaling pathway activation [10]. This activation promotes the expression of inflammatory cytokines and chemokines and triggers the inflammatory response. Peptidoglycan, as the specific ligand of NOD1 and NOD2, has been reported to be involved in atherosclerosis, contributing to the instability of atheromas [11]. Our previous results demonstrated the involvement of the NOD1/Rip2 signaling pathway in vascular restenosis following injury to rat carotid arteries and in the proliferation and phenotypic modulation of vascular smooth muscle cells. These results suggest that the activation of the NOD1/Rip2 signaling pathway may contribute to the development of atherosclerosis through a yet-to-be-determined mechanism, which has rarely been reported.

In this study, we investigated the effects of the NOD1/Rip2 signaling pathway on macrophage inflammatory activation and phenotypic variation in THP-1-derived macrophages. We investigated the functional mechanism of this signaling pathway in the formation and progression of foam cells, which could contribute to the prevention and treatment of atherosclerosis.

## 2. Materials and Methods

### 2.1. Reagents

RPMI 1640 medium was purchased from HyCLone (South Logan, UT, USA). Fetal bovine serum was purchased from Beijing Quanshijin Biotechnology (Beijing, China). ox-LDL was purchased from Guangzhou YiYuan Biotechnology (Guangzhou, China). Reverse transcription kits and real-time quantitative PCR kits were purchased from TaKaRa (Dalian, China). HiPerFect transfection reagent was purchased from Qiagen (Duesseldorf, Germany). Phorbol 12-myristate 13-acetate (PMA) was purchased from Sigma-Aldrich (St. Louis, MO, USA). Rabbit antihuman NOD1 polyclonal antibody was purchased from Abcam (Cambridge, MA, USA). A rabbit antihuman RIP2 polyclonal antibody was purchased from Santa Cruz Biotechnology (Santa Cruz, CA, USA). Rabbit antihuman β-actin monoclonal antibody was purchased from Proteintech (Wuhan, China). Protein extraction kits and BCA protein concentration determination kits were purchased from KeyGen Biotech (Hangzhou, China). Oil red O and DAPI were purchased from Sigma-Aldrich (St. Louis, MO, USA). Human MCP-1 and TNF-α ELISA kits were purchased from BioTech (Wuhan, China). FITC-conjugated antihuman CD80 antibody was purchased from BioLegend (San Diego, CA, USA). FITC-conjugated antihuman CD86 and FITC-conjugated mouse antihuman CD163 antibodies were purchased from BD Biosciences (San Jose, CA, USA).

### 2.2. Cell Culture and Differentiation

The THP-1 cell line was purchased from the Cell Bank of Shanghai Institutes for Biological Sciences (Shanghai, China). The cells were cultured in tissue culture flasks or plates in humid air with 5% CO_2_ at 37°C. THP-1 cells were suspended in fresh RPMI 1640 medium supplemented with 10% fetal bovine serum, resulting in single, round suspended cells. After two or three days, the cells were passaged once from one to two or three. After three or four passages, the cells were plated into six-well plates at a density of 1 × 10^6^/mL and stimulated with 160 nmol/L PMA. After 24 h, the cells attached to the plates, stretched into irregular or spindle shapes, and ultimately differentiated into macrophages. THP-1-derived macrophages were treated with different concentrations of ox-LDL (10, 25, or 50 mg/L) for 24 h and then incubated with 50 mg/L ox-LDL for different durations (8, 16, or 24 h).

### 2.3. Quantitative Real-Time RT–PCR Analysis

Total RNA was extracted from each sample group using TRIzol, followed by reverse transcription into cDNA via the GoScriptTM Reverse Transcription System (Promega) and PCR amplification. The PCR mixture was 20 μL in volume and contained 10 μL of SYBR Premix, 1 μL of 10 μmol/L PCR forward primer, 1 μL of 10 μmol/L PCR reverse primer, 0.4 μL of ROX, 2 μL of cDNA, and 5.6 μL of sterile distilled water. The PCR conditions included initial denaturation at 95°C for 30 s, followed by 40 cycles of denaturation at 95°C for 3 s, annealing at 55°C for 30 s, and extension at 72°C for 30 s. The amplification was conducted by real-time RT–PCR with Power SYBR Premix Ex TaqTM II in an iQ5 real-time PCR detection system with analysis software (Bio-Rad, Hercules, CA, USA). Relative gene expression was calculated via the 2^−Δ*CT*^ method. The primers (Table 1) were designed using Primer Premier Software 6.0.

### 2.4. Western Blot Analysis

The collected cells were lysed for protein extraction. The protein concentration was determined via a BCA assay according to the manufacturer's instructions. The samples were mixed with an equal volume of SDS loading buffer. After boiling, the samples were separated on a sodium dodecyl sulfate–polyacrylamide (SDS) gel at a constant current (220 mA) for 100 min and then transferred to equilibrated PVDF membranes. The membranes were incubated with a primary antibody overnight at 4 °C. Then, the membrane was washed three times for 10 min in TBS-T and incubated with the appropriate horseradish peroxidase-coupled secondary antibody for 2 h. The membranes were rinsed in TBS-T, and the signals were detected with a chemiluminescence (ECL) detection system. Band intensities were analyzed via ImageJ software (National Institutes of Health, Bethesda, MD, USA).

### 2.5. Rip2 siRNA Transfection

Rip2 siRNAs were designed according to the Rip2 mRNA sequence. Rip2 siRNA-1 (sense strand): 5′GAGAACAUUUGAAGAGAUAdTdT3′, and (antisense strand) 3′dTdTCUCUU GUAAACUUCUCUAU5′; Rip2 siRNA-2 (sense strand) 5′CAAUA UGACUCCUCCUUUA dTdT3′ and (antisense strand) 3′dTdT GUUAUACU GAGGAGGAAAU 5′; Rip2 siRNA-3 (sense strand) 5′GAAAGAGGACUAU GAACUU dTdT 3′ and (antisense strand): 3′dTdT CUUUCUCCUGA UACUUGAA 5′. Rip2 siRNAs were transfected into macrophages via the different transfection reagents at concentrations of 30, 50, and 80 nmol/L. A fluorescence microscope (Olympus Corporation, Tokyo, Japan) was used to observe the preliminary transfection efficiency. To select the most effective siRNA, the expression of Rip2 was detected via quantitative real-time PCR and Western blotting after transfection for 24 h. The most efficient siRNA was transfected into the macrophages.

### 2.6. ELISA

The culture supernatants were collected and frozen at −80°C. The original density standards were added to the EP tubes via serial dilution. All the wells were divided into blanks, standards, and samples. One hundred microliter samples were added to the wells, incubated for 90 min at 37°C, and then washed 5 times. Biotinylated antibodies were added to each well except for the blank well. After the plate was closed, the plate was incubated for 60 min at 37°C. The washing step was repeated, and the enzyme-labeled working buffer was added to each well except the blank one. Then, the plate was closed and incubated for 30 min at 37°C in the dark. The samples were washed again. Chromogen solutions were added to each well, and the samples were incubated in the dark for 15 min at 37°C. The reaction was terminated by the addition of a stop solution and gently mixing well. The color change was measured spectrophotometrically at a wavelength of 450 nm. The concentrations of the samples were then determined by comparing the OD values of the samples to those of the standard curve.

### 2.7. Fluorescence-Activated Cell Sorting (FACS)

Flow cytometry analyses were performed on a FACS Calibur flow cytometer (BD Biosciences, Franklin Lakes, NJ, USA) and analyzed using FlowJo software. Cultured cells were suspended in the indicated tubes for the expression of the cell surface markers via the use of 20 μL of FITC-conjugated monoclonal antibodies to generate a total of 100 μL of cell suspension, which was mixed well. The tubes were subsequently incubated at room temperature for 15 min in the dark. The cells were centrifuged at 2500 rpm for 3 min, and the supernatant was discarded. The pellets were suspended in 0.5 mL of PBS and analyzed on the FACS Calibur instrument.

### 2.8. Oil Red O Staining

The cells were washed 3 times in phosphate-buffered saline (PBS; 0.01 mol/L, pH = 7.4) and fixed in 4% paraformaldehyde for 10 min. The cells were subsequently stained with Oil Red O solution (0.5% in isopropanol), diluted with double distilled water at a ratio of 3:2 for 30 min at 37°C, and then washed with PBS again. Histopathological changes and cells with red-stained lipid droplets were observed and photographed via a stereomicroscope. Each cell sample was observed in at least 6 fields, and the cell count was analyzed according to the area unit via ImageJ software.

### 2.9. Statistical Analyses

Statistical analysis was conducted using SPSS 21.0 (IBM, Armonk, New York, USA). The results are expressed as the means ± SEMs. Statistical comparisons between groups were performed via ANOVA with a Holm–Sidak post-hoc test; *p* < 0.05 was considered to indicate significance.

## 3. Results

### 3.1. Expression of NOD1 and Rip2 in THP-1-Derived Macrophages After Stimulation With ox-LDL

**Figure 1:**
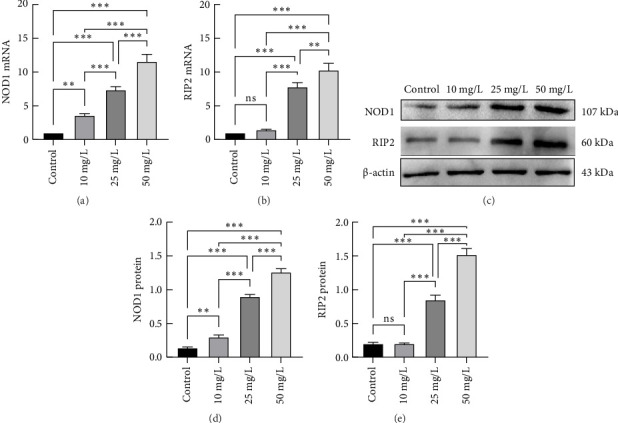
mRNA and protein expression of NOD1 and Rip2 in THP-1-derived macrophages after stimulation with different concentrations of ox-LDL. THP-1-derived macrophages were treated with increasing concentrations (10, 25, or 50 mg/L) of ox-LDL for 24 h. The expression of NOD1 and Rip2 mRNAs was measured via qRT–PCR (a and b). The protein expression of NOD1 and Rip2 was determined by Western blotting (c, d, and e). (⁣^∗^*p* < 0.05, ⁣^∗∗^*p* < 0.01 and ⁣^∗∗∗^*p* < 0.001) All results are expressed as the means ± SEMs from three independent experiments.

**Figure 2:**
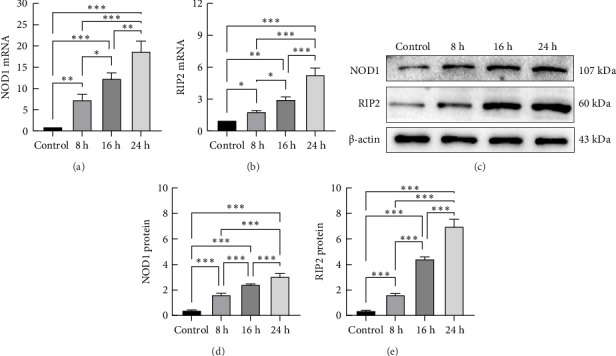
mRNA and protein expression of NOD1 and Rip2 in THP-1-derived macrophages after stimulation with ox-LDL (50 mg/L) for different durations. THP-1-derived macrophages were treated with ox-LDL (50 mg/L) for increasing durations (8, 16, and 24 h). The expression of the NOD1 and Rip2 mRNAs was measured via qRT–PCR (a and b). The protein expression of NOD1 and Rip2 was determined by Western blotting (c, d, and e) (⁣^∗^*p* < 0.05, ⁣^∗∗^*p* < 0.01, and ⁣^∗∗∗^*p* < 0.001). All results are expressed as the means ± SEMs from three independent experiments.

We used ox-LDL as an inflammatory stimulus for THP-1-derived macrophages at different concentrations (10, 25, or 50 mg/L) for 24 h. The mRNA and protein expression levels of NOD1 and Rip2 increased with increasing concentrations of ox-LDL (Figures 1(a), 1(b), 1(c), 1(d), and 1(e)). THP-1-derived macrophages were also treated with ox-LDL (50 mg/L) for different durations (8, 16, or 24 h). The expression of the NOD1 and Rip2 mRNAs and proteins increased over time (Figures 2(a), 2(b), 2(c), 2(d), and 2(e)). These results suggest that the expression of the NOD1/Rip2 signaling pathway is upregulated in THP-1-derived macrophages after inflammatory activation.

### 3.2. Secretion of TNF-α and MCP-1 in the Cell Culture Supernatants of THP-1-Derived Macrophages

**Figure 3:**
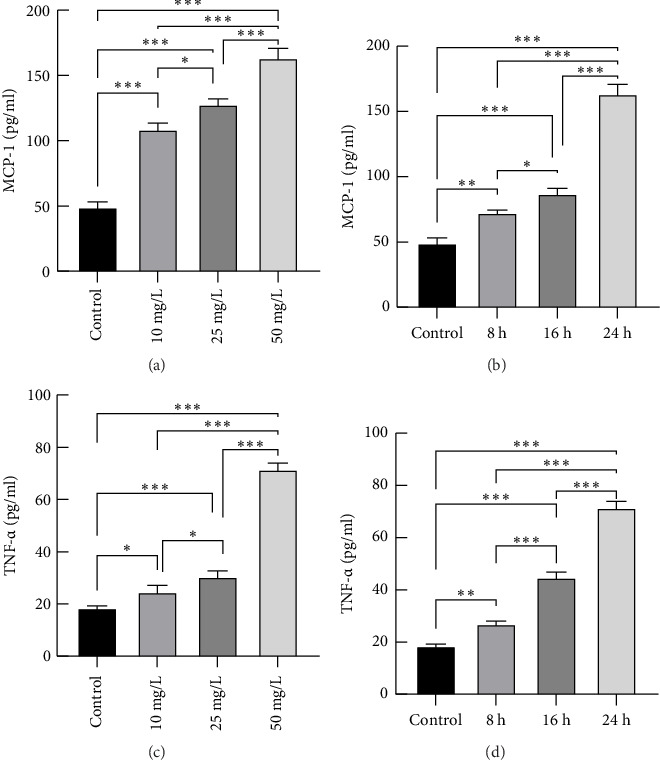
Secretion of TNF-*α* and MCP-1 in the cell culture supernatants of THP-1-derived macrophages after stimulation with ox-LDL. The secretion of TNF-*α* (c and d) and MCP-1 (a and b) was detected via ELISA. All the results are expressed as the means ± SEMs from three independent experiments (⁣^∗^*p* < 0.05, ⁣^∗∗^*p* < 0.01, and ⁣^∗∗∗^*p* < 0.001).

The concentrations of TNF-α and MCP-1 in the cell culture supernatants were determined via ELISA. Ox-LDL induced the secretion of TNF-α and MCP-1 in a dose- and time-dependent manner in THP-1-derived macrophages (Figure 3), indicating that TNF-α and MCP-1 secretion increases in THP-1-derived macrophages after activation.

### 3.3. Effects of Rip2 siRNA on THP-1-Derived Macrophages

**Figure 4:**
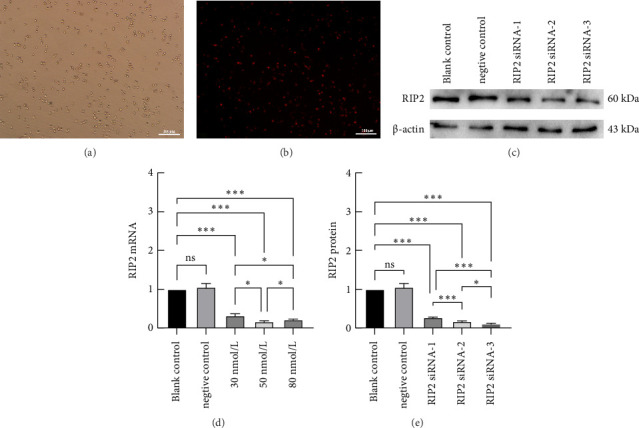
Effects of Rip2 siRNA on THP-1-derived macrophages. Rip2 siRNA was transfected into macrophages. The transfection efficiency was observed under a fluorescence microscope (a) in the same visual field preliminary (b) and an optical microscope. The Rip2 siRNA was transfected into cells at final concentrations of 30, 50, and 80 nmol/L, and the most effective siRNA concentration was measured via qRT–PCR (d). To select the most effective siRNA, the expression of the Rip2 protein was determined via western blotting (c and e) (⁣^∗^*p* < 0.05, ⁣^∗∗^*p* < 0.01, and ⁣^∗∗∗^*p* < 0.01). All the results are expressed as the means ± SEMs from three independent experiments.

THP-1-derived macrophages were transfected with Rip2 siRNA for 24 h. The fluorescence microscopy picture (Fig.  4(b)) showed that most of the cells emitted red fluorescence in the same field of vision compared to an ordinary light microscope image (Fig.  4(a)), indicating that most cells were successfully transfected with siRNA. Different concentrations of Rip2 siRNA (30, 50, and 80 nmol/L) were transfected into the macrophages. Real-time PCR revealed that 50 nmol/L was the most efficient concentration (Figure 4(d)); therefore, 50 nmol/L was used in the subsequent experiments. Three siRNAs were designed, and Western blot analysis revealed that Rip2 siRNA-3 had the most potent suppressive effect on Rip2 (Figure 4(c), and 4(e)).

### 3.4. Effects of Rip2 siRNA on Foam Cell Formation and TNF-α and MCP-1 Secretion in THP-1-Derived Macrophages After Stimulation With Ox-LDL

**Figure 5:**
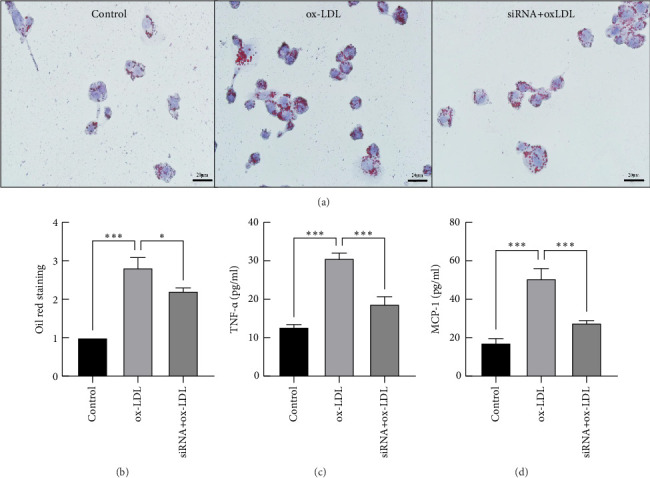
Effects of Rip2 siRNA on foam cell formation and TNF-*α* and MCP-1 expression in THP-1-derived macrophages stimulated with ox-LDL. THP-1-derived macrophages were transfected with Rip2 siRNA, and then, the cells were treated with 50 mg/L ox-LDL for 24 h. Oil Red O staining was employed to visualize foam cell formation (a and b), and ELISA was used to detect TNF-*α* and MCP-1 secretion in the cell culture supernatant (c and d) (⁣^∗^*p* < 0.05, ⁣^∗∗^*p* < 0.01, and ⁣^∗∗∗^*p* < 0.001) All the results are expressed as the means ± SEMs from three independent experiments.

After being transfected with the most efficient Rip2 siRNA, THP-1-derived macrophages were treated with 50 mg/L ox-LDL for 24 h. Oil Red O staining was employed to visualize foam cell formation. The formation of foam cells significantly increased in the ox-LDL group. Conversely, RIP2 siRNA significantly inhibited ox-LDL-induced foam cell formation (Figures 5(a) and 5(b)). ELISA was used to determine TNF-α and MCP-1 secretion in the cell culture supernatant. The results showed that Rip2 siRNA suppressed the secretion of TNF-α and MCP-1 by THP-1-derived macrophages after stimulation with ox-LDL (Figures 5(c) and 5(d)).

### 3.5. Effects of Rip2 siRNA on M1 and M2 Marker Expression in THP-1-Derived Macrophages After Stimulation With Ox-LDL

**Figure 6:**
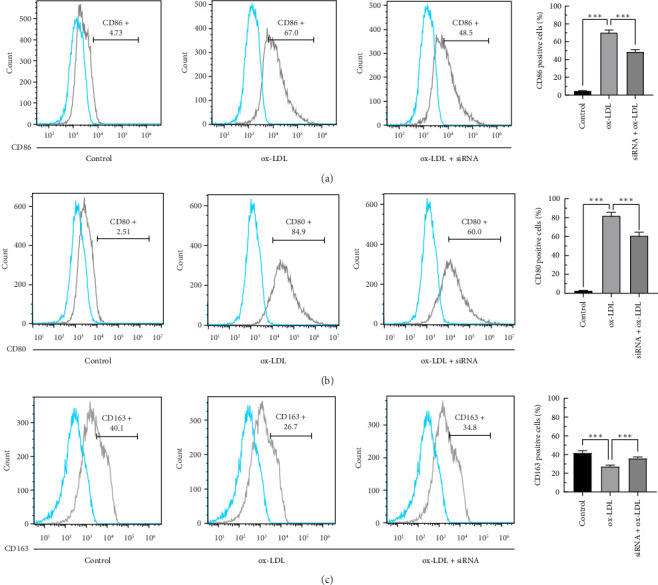
Effects of Rip2 siRNA on CD86, CD80, and CD163 expression in THP-1-derived macrophages stimulated with ox-LDL. FACS was used to detect the expression of the membrane molecules CD86, CD80, and CD163 (a, b, and c). All the results are expressed as the means ± SEMs from three independent experiments (⁣^∗^*p* < 0.05, ⁣^∗∗^*p* < 0.01, and ⁣^∗∗∗^*p* < 0.001).

**Figure 7:**
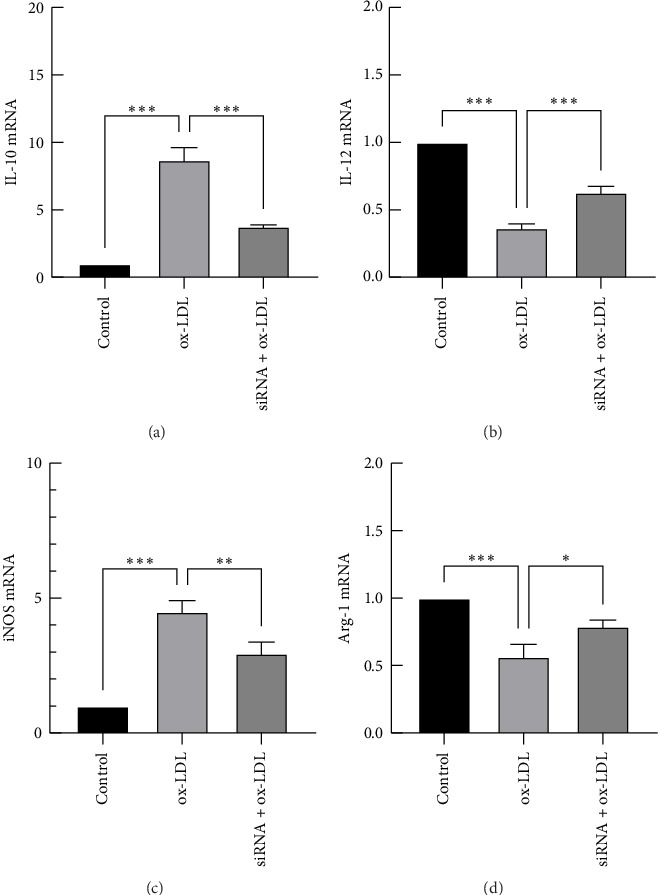
Effects of Rip2 siRNA on IL-10, IL-12, iNOS, and Arg-1 expression in THP-1-derived macrophages stimulated with ox-LDL. qRT–PCR was used to measure the mRNA expression of IL-10, IL-12, iNOS, and Arg-1 (a, b, c, and d). All the results are expressed as the means ± SEMs from 3 independent experiments (⁣^∗^*p* < 0.05, ⁣^∗∗^*p* < 0.01, and ⁣^∗∗∗^*p* < 0.001).

FACS was used to detect the membrane molecules CD86, CD80, and CD163; qRT–PCR was used to measure the mRNA expression of IL-10, IL-12, iNOS, and Arg-1. The expression of CD163, IL12, and Arg-1 was decreased in THP-1-derived macrophages after stimulation with ox-LDL (Figures 6(c), 7(b), and 7(d)), but the expression of CD86, CD80, IL-10, and iNOS was increased (Figures 6(a), 6(b), 7(a), and 7(c)). Furthermore, compared with ox-LDL, RIP2 siRNA upregulated the expression of CD163, IL12, and Arg-1 but downregulated the expression of CD86, CD80, IL12, and Arg-1. Rip2 siRNA could inhibit the effect induced by ox-LDL. CD86, CD80, IL-10, and iNOS are markers of M1 macrophages, and CD163, IL12, and Arg-1 are markers of M2 macrophages. These results suggest that the phenotype of the macrophages changes from M1 to M2 after stimulation with ox-LDL and that Rip2 siRNA inhibits this macrophage polarity switching.

## 4. Discussion

The innate immune system is known to contribute to the development and progression of atherosclerosis. The initiation and progression of atherosclerosis depend on local inflammation and the accumulation of lipids in the vascular wall [12, 13]. Ox-LDL, an atherosclerosis-related antigen, is the most important factor leading to atherosclerosis. Macrophages, which take up ox-LDL to form foam cells and release inflammatory cytokines, chemokines, and agonists/inhibitors of enzymes, are considered key factors in atherosclerosis development and progression. Statins effectively reduce ox-LDL levels and lower inflammatory responses, thereby contributing to the treatment of CAD [14]. In addition to drugs, bariatric surgery also has a beneficial effect on ox-LDL [15].

As an important member of the NLR family, NOD1 plays an essential role in immune surveillance and cell defense. Our data revealed that the expression of NOD1 and Rip2 at both the mRNA and protein levels in THP-1-derived macrophages increased in a dose-dependent and time-dependent manner after being stimulated with ox-LDL. Moreover, after stimulation with ox-LDL, the secretion of TNF-α and MCP-1 in the macrophage culture supernatant increased, but after Rip2 gene silencing, the secretion of TNF-α and MCP-1 decreased. Ox-LDL can trigger an inflammatory response by activating Toll-like receptors (TLRs), which are damage-associated molecular patterns [16, 17]. Ox-LDL plays a vital role in the development and progression of atherosclerosis. In this study, we showed that macrophages could be activated by ox-LDL. After activation, macrophages can produce more inflammatory factors, and this process can be blocked by inhibiting the NOD1/Rip2 signaling pathway. These results suggest that the NOD1/Rip2 signaling pathway may be involved in macrophage inflammatory activation.

There are few studies on the role of the NOD1 signaling pathway in the development of atherosclerosis. Kanno et al. [18] reported that the atherosclerosis process was accelerated by long-term feeding of ApoE^−/−^ mice with FK565 (NOD1 ligand). In addition, NOD1 knockout reduced the size of atheromas in ApoE^−/−^ mice. González-Ramos et al. [19] demonstrated that the deletion or inhibition of NOD1 promotes plaque stability and mitigates atherothrombosis in advanced atherogenesis. These findings suggest that NOD1 and the NOD1/Rip2 signaling pathway may be involved in the development of atherosclerosis. However, the underlying mechanisms remain unknown.

Activated NOD1 can recruit downstream Rip2 and activate IKK, which degrades IKB-α, an inhibitor of NF-*κ*B. NF-*κ*B then translocates into the nucleus and activates the transcription of NF-*κ*B-dependent genes, such as TNF-α and MCP-1 [20]. Similarly, an in vitro study in mouse macrophages cocultured with ES from adult *Toxocara canis* revealed that the NOD1-RIP2-NF-*κ*B signaling pathway was expressed at both the transcriptional and translational levels after 9 h of incubation. The production of proinflammatory cytokines, including TNF-α, IL-1β, and IL-6, released by stimulated macrophages is modulated [21]. This study revealed that TNF-α and MCP-1 could be induced by ox-LDL, but after Rip2 gene silencing, this effect was inhibited. TNF-α can promote vascular endothelial cell and smooth muscle cell secretion of adhesion factors and fibroblast promotion, and macrophages secrete interleukin-1, interleukin-6, and TNF-α, which are widely involved in inflammation and macrophage migration and proliferation and play key roles in atherosclerosis [22]. MCP-1 can mediate the infiltration of monocytes into endothelial cells, accelerate lipoprotein uptake in gaps, promote foam cell formation, and enhance monocyte migration and adhesion, which accelerates the process of atherosclerosis [23]. Thus, the NOD1/Rip2 signaling pathway could contribute to atherosclerosis development by regulating macrophage inflammatory activation. Notably, Rip2 and Nod1 may play additional roles in cardiovascular disease in addition to inflammation because they mediate angiogenic activity in endothelial cells.

Macrophages are the major inflammatory cells involved in the progression of atherosclerosis. Macrophage polymerization is currently under intensive study. The polarized Th1/Th2 macrophages are divided into classically activated macrophages (M1 macrophages) and alternatively activated macrophages (M2 macrophages) [24]. Recently, in vivo studies confirmed that differentiated M1/M2 macrophage subsets can switch between M2 and M1 phenotypes during atherosclerotic plaque formation [25]. M1 macrophages predominantly elicit proinflammatory responses, synthesizing inflammatory factors, including TNF-α and IL-6, as well as markers such as MCP-1 [26]. During the progression of AS inflammation, macrophages mainly polarize toward the M1 phenotype and express high levels of CD86, CD80, iNOS, IL-12, and other proinflammatory factors, thereby promoting AS development [27]. Conversely, in the resolution phase of inflammation, M2 macrophages dominate, expressing high levels of the mannose receptor CD163 and releasing Arg-1, IL-10, and other cytokines. These actions help mitigate the inflammatory response, maintain vascular homeostasis, and exert antiatherosclerotic effects [28]. This study revealed that incubation with ox-LDL decreased the expression of M2 markers and increased the expression of M1 markers, which indicated that ox-LDL could lead to the transformation of M1 macrophages. Luo et al. [29] reported that M1 macrophages are polarized in response to ox-LDL and cytokines and then accelerate plaque progression and vulnerability by releasing proinflammatory cytokines, including high levels of IL-1β, IL-6, and TNF-α. Research by Wu et al. [30] also yielded similar results. In vitro and in vivo studies by Song et al. [31] confirmed that the overexpression of miR-30a-5p reduced the M1/M2 macrophage ratio, leading to a reduction in the levels of proinflammatory factors, such as IL-6 and TNF-α, in the plasma. Bosmans et al. [32] reported that targeted knockout of CD40 led to the upregulation of genes related to M2 macrophage markers, which subsequently resulted in a significant decrease in the expression of inflammation-related genes. These findings correlated with improvements in AS plaque severity and stability. This study investigated the impact of Rip2 siRNA on the expression of M1 and M2 markers and demonstrated that Rip2 siRNA suppressed the effects of ox-LDL. These findings also indicate that the NOD1/RIP2 signaling pathway regulates the polarization of macrophages, promoting M1 macrophage polarization and inhibiting M2 macrophage polarization, thereby suppressing inflammation and affecting macrophage activation.

## 5. Conclusion

**Figure 8:**
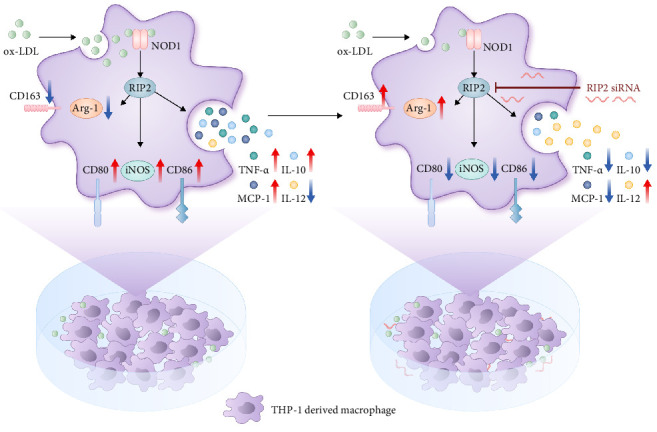
Effects of NOD1/Rip2 on macrophage inflammatory activation and polarization in THP-1-derived macrophages stimulated with ox-LDL. After exposure to ox-LDL, the concentrations of TNF-*α* and MCP-1 increased. Furthermore, ox-LDL downregulated the expression of M2 macrophage markers CD163, IL-12, and Arg-1 and upregulated the expression of M1 macrophage markers CD86, CD80, IL-10, and iNOS. The inhibition of Rip2 by siRNA reversed these effects and prevented the formation of foam cells.

Our research confirms the important role of the NOD1/Rip2 signaling pathway in the inflammatory activation of macrophages. This effect is likely achieved through altering macrophage polarization, which may be an important mechanism for the NOD1/Rip2 signaling pathway in foam cell formation and development (Figure 8). These findings provide new data for identifying the role of immune responses in atherosclerosis and offer new clues for the prevention of atherosclerosis in the future.

## Figures and Tables

**Table 1:** Primers used for quantitative real-time PCR.

Gene	Primer sequence (5′ to 3′)	Product (bp)
*β*-actin	F: GGGAAATCGTGCGTGACATT R: GGAACCGCTCATTGCCAAT	150
NOD1	F: GTATCTCGCCCTGGCTGTGA R: ATGCCGTTGGACGCAAGA	151
RIP2	F: GCTGCATCACTGTCCTGGAA R: CCAGGCTGCAGACGTTCTG	149
IL-10	F: TCCTTGTGGCTACCCTGGTCCT R: TCCTTGTGGCTACCCTGGTCCT	114
IL-12	F: GGCGCTGTCATCGATTTCTTCC R: GGCGCTGTCATCGATTTCTTCC	114
iNOS	F: CTGCAGCACTTGGATCAGGAACCTG R: GGAGTAGCCTGTGTGCACCTGGAA	311
Arg-1	R: GGAGTAGCCTGTGTGCACCTGGAA R: GGAGTAGCCTGTGTGCACCTGGAA	137

## Data Availability

The data that support the findings of this study are available on request from the corresponding author, upon reasonable request.
